# Breathing for Two: Asthma Management, Treatment, and Safety of Pharmacological Therapy during Pregnancy

**DOI:** 10.3390/medicines11070018

**Published:** 2024-09-05

**Authors:** Jovan Javorac, Dejan Živanović, Biljana Zvezdin, Vesna Mijatović Jovin

**Affiliations:** 1 Department of Internal Medicine, Faculty of Medicine, University of Novi Sad, 21000 Novi Sad, Serbia; biljana.zvezdin@mf.uns.ac.rs; 2 Institute for Pulmonary Diseases of Vojvodina, 21204 Novi Sad, Serbia; 3 Faculty of Medicine, University of Novi Sad, 21000 Novi Sad, Serbia; dejan.zivanovic@mf.uns.ac.rs; 4 Department of Psychology, College of Human Development, 11000 Belgrade, Serbia; 5 Department of Pharmacology, Toxicology and Clinical Pharmacology, Faculty of Medicine, University of Novi Sad, 21000 Novi Sad, Serbia; vesna.mijatovic-jovin@mf.uns.ac.rs

**Keywords:** asthma, pregnancy, treatment, safety

## Abstract

The primary objectives of asthma management during pregnancy are to achieve adequate symptom control, reduce the risk of acute exacerbations, and maintain normal pulmonary function, all of which contribute to ensuring the health and well-being of both the mother and the baby. The Global Initiative for Asthma (GINA) recommends that pregnant women with asthma continue using asthma medications throughout pregnancy, as the benefits of well-controlled asthma for both the mother and fetus outweigh the potential risks of medication side effects, poorly controlled asthma, and exacerbations. The classification of asthma medications by the US Food and Drug Administration (FDA) into categories A, B, C, D, and X is no longer applied. Instead, the potential benefits and risks of each medication during pregnancy and lactation are considered individually. The use of medications to achieve good asthma control and prevent exacerbations during pregnancy is justified, encompassing inhaled corticosteroids (ICS), some leukotriene receptor antagonists (LTRA), short-acting beta-2 agonists (SABA), long-acting beta-2 agonists (LABA), short-acting muscarinic antagonists (SAMA), long-acting muscarinic antagonists (LAMA), and, recently, biological therapies, even in the absence of definitive safety data during pregnancy.

## 1. Introduction

Asthma is one of the most common chronic diseases that can complicate pregnancy, and it is the most prevalent chronic pulmonary disease during pregnancy [1]. Approximately 8% to 13% of pregnant women have asthma [2], with a rising prevalence in recent years, likely due to the increasing prevalence of asthma among young women. Among pregnant women with asthma, about 19% have severe asthma and 16% have poorly controlled asthma [3]. Some estimates suggest that in about one-third of pregnant women with asthma, symptoms may worsen during pregnancy, and in another third, symptoms may improve, while in the remaining third, symptoms may not significantly change during pregnancy compared to the usual course of the disease [4]. A recent study provided slightly different results, indicating that asthma symptoms worsened in 40% of the monitored pregnant women, while in the remaining 60%, there was no change in symptoms during pregnancy [5]. When exacerbations of asthma occur, they most commonly occur in the second and third trimesters, with the highest incidence in the sixth month of pregnancy [6]. Given the variability and unpredictability of these symptom changes, it is crucial that asthma is well-controlled during pregnancy and that acute exacerbations are prevented. Poor asthma control and exacerbations during pregnancy are associated with adverse outcomes for the pregnancy, the mother, and the fetus. Pregnancy complications such as spontaneous abortion, antepartum and postpartum hemorrhage, placental abruption, and placenta previa are more common in pregnant women with asthma compared to those without asthma. This is also true for fetal complications (congenital malformations, preterm birth, small for gestational age, low birth weight, increased perinatal mortality, higher incidence of asthma, and other respiratory, infectious, dermatological, and hematological diseases in childhood), as well as maternal complications (increased risk of gestational hypertension, preeclampsia, eclampsia, gestational diabetes, obesity, and pulmonary thromboembolism) [1,3,4,7]. Additionally, there is evidence suggesting that the frequency of cesarean deliveries is higher among pregnant women with asthma, especially those with severe asthma, compared to those without asthma [8]. On the other hand, it is estimated that globally, up to a quarter of pregnant women with asthma discontinue their prescribed medication during pregnancy due to fears of potential negative effects on the fetus [9], thereby putting both themselves and the fetus at risk. Considering this, the aim of this review is to critically examine current recommendations for the management and treatment of asthma during pregnancy, including the management of acute asthma exacerbations.

## 2. Asthma Management during Pregnancy

Managing asthma during pregnancy, similar to non-pregnant periods, involves an individualized approach to assess asthma control (determined by symptom control and the risk of future adverse events such as asthma exacerbations, development of fixed airway obstruction, and medication side effects), addressing therapeutic issues, and evaluating comorbidities [4]. Strong evidence indicates that if asthma is well-controlled during pregnancy, the risks of maternal or fetal complications are minimal or nonexistent. If pregnant women continue using their prescribed asthma therapy, the severity of asthma during pregnancy is similar to its severity before pregnancy [3]. The 2024 guidelines from the Global Initiative for Asthma (GINA) emphasize the importance of maintaining asthma control during pregnancy to minimize risks [4]. Despite this, research data indicate that up to 65% of pregnant women have poorly controlled asthma, a similar percentage do not use their inhalers correctly during pregnancy, and only about 13% have a written asthma action plan [10]. Key aspects of asthma control during pregnancy are discussed below.

### 2.1. Assessment and Monitoring of Asthma Control

The assessment of symptom control involves taking a detailed medical history at each visit during pregnancy. Standardized questionnaires, such as the Asthma Control Test (ACT) or the Asthma Control Questionnaire (ACQ), can be helpful in assessing symptom control [1]. Pulmonary function tests, including spirometry or peak expiratory flow (PEF) measurements, should be a part of routine asthma monitoring during pregnancy as well [4]. Despite the diaphragm elevation due to fetal growth, forced vital capacity (FVC), forced expiratory volume in one second (FEV1), the FEV1/FVC ratio, and PEF values do not change compared to pre-pregnancy levels [11]. Therefore, any decline in lung function during pregnancy should be taken with caution, as it does not represent a physiological effect of pregnancy. In uncomplicated pregnancies, monthly spirometry is safe for the mother, pregnancy, and fetus, but bronchoprovocation tests are not recommended [6]. A prospective study examined the effect of measuring the fraction of exhaled nitric oxide (FeNO) on asthma control during pregnancy, finding that exacerbation rates were lower and that quality of life was better in women whose FeNO levels were monitored [12]. The development of telemedicine and modern communication technologies allows for remote monitoring pregnant asthma patients, enabling remote symptom monitoring, virtual consultations, and automated medication reminders, reducing the burden of traveling to healthcare facilities as well as improving symptom control, patients’ quality of life, and adherence to treatment plans [13,14].

### 2.2. **Patient Education**

Asthma control should be assessed at every pre-conception visit and during pregnancy planning, with regular check-ups recommended during pregnancy. For women with moderate to severe asthma or those with frequent exacerbations, monthly check-ups and spirometry may be recommended. For those with mild, well-controlled asthma, the frequency of visits may be adjusted based on individual needs and clinical judgment [4]. Good asthma control before conception is associated with better pregnancy outcomes for both the mother and the fetus [1]. Education should be a fundamental part of each check-up, focusing on the proper and regular use of prescribed inhalers. Pregnant women should be educated on having an asthma action plan, detailing steps to take if symptoms worsen and when to seek medical help. Emergency plans should be discussed regularly and be easily accessible [4]. The essential elements of an asthma action plan, such as monitoring symptoms, using medications, and knowing when to seek medical help, stay consistent before and during pregnancy. However, the plan typically needs adjustments during pregnancy, including selecting safer medication alternatives, increasing lung function monitoring (with more frequent peak flow readings), offering guidance on distinguishing between asthma symptoms and pregnancy-related symptoms like dyspnea, and modifying the emergency plan to account for when to seek immediate medical attention [15]. Shared decision-making between the patient and healthcare team is crucial, educating the pregnant woman about the safety of asthma medications during pregnancy and the potential teratogenic effects of these drugs to prevent the discontinuation of prescribed medications without consulting a healthcare professional.

### 2.3. Exposure to Allergens and Asthma Triggers

Identifying and avoiding asthma triggers is essential. Pregnant women should be advised on lifestyle modifications and avoiding common allergens, such as pet dander, pollen, mold, and house dust mites. Reducing exposure to environmental factors that can worsen asthma symptoms, like tobacco smoke, air pollution, perfumes, and other volatile chemicals, is also important [1]. Immunotherapy can continue during pregnancy if started before conception, but initiating it during pregnancy is not recommended [3]. Pregnant women with asthma are advised to receive the influenza vaccine to reduce the risk of exacerbations associated with flu, which can be particularly concerning during pregnancy [4]. The influenza vaccine is considered to be safe and effective throughout all trimesters and should be part of routine prenatal care of all pregnant women. The vaccine can be administered in the first trimester if the flu season is approaching or ongoing, while pregnant women in their second and third trimesters are also strongly advised to receive the vaccine if they have not already [16].

### 2.4. As Sessment of Comorbid Conditions

Comorbidities can significantly affect asthma control and pregnancy outcomes. Strong evidence links obesity, diabetes, hypertension, depression, anxiety, and allergic rhinitis with asthma control during pregnancy, while evidence is weaker for gastroesophageal reflux disease (GERD), iron deficiency, and vitamin D deficiency [2].

Asthma is more common in obese women, and pregnant women with asthma have higher obesity rates than those without asthma [17,18,19]. One study showed that the risk of asthma exacerbation during pregnancy is twice as high in obese pregnant women compared to those with normal weight [20]. Children of obese pregnant women are at higher risk of developing asthma in childhood [4].

Pregnant women with asthma are at higher risk of developing gestational diabetes, particularly if asthma is inadequately treated and controlled. The risk may be further influenced by the type of asthma medications used. While inhaled corticosteroids (ICS) are generally associated with a lower risk of systemic side effects and are not generally associated with gestational diabetes, the use of oral corticosteroids (OCS) can increase the risk of gestational diabetes [19]. Both maternal asthma and gestational diabetes are linked to adverse pregnancy outcomes, such as preterm birth, the need for neonatal intensive care, and low birth weight [2,21].

Pregnant women with asthma are at increased risk of developing pregnancy-induced hypertension and preeclampsia compared to those without asthma [21]. Preeclampsia is also a risk factor for asthma in the newborn [22].

Asthma patients have higher prevalence rates of depression and anxiety compared to those without asthma, including pregnant women. Studies indicate that pregnant women with depression and anxiety more frequently have poorly controlled asthma (53%) compared to those without these conditions (33%) [23]. They also experience higher rates of adverse pregnancy outcomes, such as preeclampsia, eclampsia, labor interventions, and reduced neonatal birth weight [24].

The prevalence of allergic rhinitis is high among pregnant women with asthma. Those with both asthma and allergic rhinitis have higher anxiety levels, poorer asthma control, and lower asthma-related quality of life compared to those without rhinitis [2]. Therefore, educating pregnant women with allergic asthma and rhinitis on lifestyle modifications to reduce exposure to potential allergens is crucial. However, it is important to distinguish allergic rhinitis from rhinitis of pregnancy, which is a common condition characterized by nasal congestion, rhinorrhea, and sneezing that is not related to allergic reactions. While both allergic rhinitis and rhinitis of pregnancy can co-occur, they have different underlying mechanisms and management approaches [25].

GERD is a common comorbidity in asthma and pregnant women due to elevated progesterone levels that can relax the lower esophageal sphincter. GERD presence in pregnant women with asthma can worsen asthma symptoms [3]. However, few studies have examined the effect of GERD on asthma symptoms in pregnant women, necessitating further research in this area.

## 3. Pharmacological Treatment of Asthma during Pregnancy and Its Safety

The primary objectives of managing asthma during pregnancy are to achieve adequate symptom control, reduce the risk of acute exacerbations, and maintain normal pulmonary function [6]. GINA recommends that pregnant women with asthma continue their asthma medications throughout pregnancy, as the benefits of well-controlled asthma during pregnancy for both the mother and the fetus far outweigh the potential teratogenic effects of the medications, poorly controlled asthma, and asthma exacerbations [4]. Consequently, the previous Food and Drug Administration (FDA) classification of drugs into categories A, B, C, D, and X is no longer used for asthma medications during pregnancy. Instead, the potential benefits and risks of each drug during pregnancy and lactation are evaluated individually [26].

The use of medications to achieve good asthma control and prevent exacerbations is justified even if the safety of these medications during pregnancy has not been conclusively proven. This includes ICS, leukotriene receptor antagonists (LTRA), short-acting beta-2 agonists (SABA), long-acting beta-2 agonists (LABA), short-acting muscarinic antagonists (SAMA), long-acting muscarinic antagonists (LAMA), systemic steroids, and, more recently, biological agents [4]. Effective asthma management during pregnancy involves not only the appropriate selection of medications, but also patient education, ensuring proper inhalation techniques, advising on the regular use of medications (given the tendency of pregnant women to reduce medication use during pregnancy), and treating comorbid conditions.

GINA recommendations particularly emphasize the safety of ICS, which are the cornerstone of asthma management. Strong evidence suggests that ICS use reduces the rate of asthma exacerbations during pregnancy, while their discontinuation significantly increases the risk of exacerbations [4]. Additionally, ICS use during pregnancy has not been associated with an increased risk of congenital heart defects, other congenital malformations, preterm birth, or low birth weight [3]. Randomized clinical trials involving pregnant women during early pregnancy (12–20 weeks) demonstrated a protective effect of ICS on the risk of asthma in their offspring. Given these benefits, GINA does not recommend any reduction in asthma therapy until after pregnancy, and ICS should not be excluded from therapy during pregnancy planning or throughout the pregnancy [4]. Preferred ICS options during pregnancy include budesonide, beclomethasone, and fluticasone propionate, as there is insufficient evidence regarding the safety of other ICS such as ciclesonide, mometasone, or fluticasone furoate [6]. Historically, the FDA classified budesonide as Category B, indicating that it was to be considered relatively safer during pregnancy compared to other ICSs that were classified as Category C. With the introduction of the new FDA Pregnancy and Lactation Labeling Rule (PLLR), the categories A-X are no longer used. Instead, medication safety is now evaluated based on current evidence and detailed labeling. According to the latest evidence, budesonide is generally preferred over other ICS for pregnant women due to its well-established safety profile [1,11]. However, the choice of ICS should be individualized, considering the specific clinical needs of the patient and the available safety data for each medication.

GINA also provides recommendation for the safe use of standard doses of LABA in combination with ICS during pregnancy, including ICS-formoterol combination used as a controller and reliever therapy [4]. Experimental animal studies and observational studies in humans have not established an association between formoterol and salmeterol use and adverse fetal outcomes [3]. However, less evidence exists for SABA use as a quick-relief therapy, although it is unlikely to cause structural fetal defects. Nevertheless, systemic absorption of SABA can lead to maternal and fetal tachycardia and maternal and neonatal hypoglycemia; thus, excessive use is not recommended [26]. High doses of ipratropium bromide (SAMA) used in acute asthma attacks have not demonstrated fetal toxicity in animal models, with minimal effects on maternal and fetal heart rate. There is minimal evidence of safety risks for tiotropium bromide (LAMA), but their use is not restricted when adequate control is not achieved with ICS-LABA therapy [6]. This can similarly be said for other LAMA that are usually used in combination therapy, such as umeclidinium and glycopyrronium, which should be used during pregnancy only if the expected benefit to the mother outweighs the potential risk to the fetus.

LTRA can be used as a second-line maintenance therapy during pregnancy. Although evidence of their use during pregnancy is limited, some studies suggest that they are safe for the fetus and mother [2] and that the therapeutic benefits of montelukast during pregnancy outweigh the potential risks to the fetus. The use of zileuton, however, is not recommended during pregnancy due to a lack of safety data and the need for frequent liver function monitoring during its use [11].

Systemic corticosteroid use during pregnancy should be minimized and reserved for treating acute exacerbations and severe asthma, with the benefits outweighing the potential risks to the fetus, such as congenital malformations (e.g., cleft palate), preterm birth, low birth weight, and preeclampsia [3,8]. When it is necessary to include them in the therapy, healthcare providers should closely monitor their effects.

Evidence regarding the use of biological therapy for severe asthma during pregnancy is still limited. Decisions about continuing or initiating biological therapy during pregnancy should be based on consensus between the patient and the healthcare provider, considering the potential risks to the fetus and the risks of uncontrolled asthma for the mother and fetus. Although all biological agents used in asthma treatment are IgG antibodies that cross the placenta to varying degrees, there is no evidence of their teratogenic effects [6]. One study found that omalizumab use during pregnancy did not result in congenital malformations [27], but its initiation during pregnancy is not recommended—only continuation in women already receiving it before pregnancy is recommended. Animal studies have shown that benralizumab, mepolizumab, and reslizumab, as well as dupilumab and tezepelumab, do not cause congenital malformations or reduced fetal body weight, although human studies are lacking [3].

The use of antihistamines, intranasal corticosteroids (particularly budesonide), and topical corticosteroid creams for associated allergic conditions, such as allergic rhinitis and eczema, is safe during pregnancy and lactation [28,29]. First-generation antihistamines can cross the blood–brain barrier and may transfer to the infant through breast milk, potentially causing irritability, drowsiness, and sleep disturbances. Therefore, breastfeeding mothers are generally advised to avoid these medications. If antihistamine treatment is needed, second-generation antihistamines, which are less likely to affect the infant’s central nervous system, are preferred [30]. Local vasoconstrictors for treating concomitant allergic rhinitis can be used during pregnancy following standard dosing guidelines, but oral decongestants (such as phenylephrine and pseudoephedrine) should be avoided, especially during the first trimester, due to potential fetal ischemia and congenital malformations from systemic vasoconstriction [1]. Subcutaneous or sublingual immunotherapy initiation during pregnancy is not recommended due to the risk of severe allergic reactions, although therapy can continue if it was started before pregnancy and is well-tolerated by the patient. Although methylxanthines are no longer recommended for asthma treatment, their use during pregnancy and lactation is safe, but frequent monitoring of drug levels in the blood is necessary [6].

In line with these considerations, the European Respiratory Society (ERS) and the Thoracic Society of Australia and New Zealand (TSANZ) issued a joint statement in 2020 on the safety of medications for respiratory diseases during pregnancy and lactation [26]. This document no longer uses the FDA drug classification categories A–X, but instead classifies each drug into one of three categories based on the benefits and risks (Table 1):

- Compatible with use during pregnancy and/or lactation: There is sufficient evidence of the drug’s use during pregnancy, indicating very low or no risk to the embryo/fetus.
- Probably safe for use during pregnancy and/or lactation: There is limited research experience, but the drug’s characteristics suggest low risk to the embryo/fetus.
- Possibly safe for use during pregnancy and/or lactation: Drugs in this category should be used as a second-line therapy if safer options are ineffective; the direct benefits to the mother are likely to outweigh the potential risks to the embryo/fetus, although the exact risks are unknown.

## 4. Management of Acute Asthma Exacerbations during Pregnancy

Asthma exacerbations are common during pregnancy, particularly in the first and second trimesters [4]. The incidence of acute exacerbations during pregnancy varies by geographic region, ranging from 20% to 60%, with 10 to 40% of exacerbations requiring hospital treatment [31]. Acute asthma exacerbations during pregnancy are associated with a higher occurrence of congenital malformations if they occur in the first trimester, as well as being associated with preterm birth, being small for gestational age, low birth weight, preeclampsia, and an increased rate of spontaneous abortions [6].

The most common triggers for acute asthma exacerbations during pregnancy are viral infections. Based on recent evidence, rhinovirus is now recognized as one of the most common viral triggers for acute asthma exacerbations, including during pregnancy [32]. The increased susceptibility of pregnant women to viral infections is explained by reduced cellular immunity [6]. Additionally, hormonal and, to a lesser extent, mechanical changes during pregnancy affecting pulmonary ventilation may predispose pregnant women to a higher risk of acute asthma exacerbations [4]. These changes include the following:

- Increased oxygen consumption and accelerated metabolic processes by about 20%, leading to an increase in minute ventilation (up to 20–40%) [1].
- Elevation of the diaphragm due to uterine expansion, resulting in a decrease in total lung capacity, expiratory reserve volume, residual volume, and functional residual capacity, with an increase in tidal volume (up to 40%) and unchanged FVC, FEV1, and PEF [1,6].
- Increased progesterone levels that stimulate the respiratory center in the medulla oblongata, causing hyperventilation with resulting hypocapnia and transient respiratory alkalosis, which can lead to the onset of “physiological dyspnea in pregnancy”, which occurs in a quarter of all pregnant women during early pregnancy, and weakening local anti-inflammatory reactions in the bronchial mucosa [33].

A recently published systematic review identified maternal risk factors associated with an increased incidence of asthma exacerbations during pregnancy: the presence of moderate-to-severe asthma in the pregnant woman, depression and anxiety, obesity, a personal history of multiple deliveries, older maternal age (over 35 years), exposure to tobacco smoke, and African American descent [34]. Another important factor contributing to exacerbations and poor asthma control is the fear of pregnant women about taking the recommended medications upon learning that they are pregnant due to concerns about possible harmful (teratogenic) effects of the drugs on the course of the pregnancy and the fetus itself [31]. Previous research has shown that the rate of regular use of inhalers for disease control can decrease by 17–30% during the first trimester of pregnancy, with the most common reasons being a lack of information and concern about the safety of using these drugs during pregnancy, as well as a desire for a “natural” course of pregnancy [6]. In the study by Powell et al. [35], 42% of pregnant women with asthma believed there was a risk of teratogenic effects from OCS, 12% for ICS, and 5% of women believed there was a risk of teratogenic effects from using short-acting β-agonists. Although taking recommended medications during pregnancy is of invaluable importance for the health of both the pregnant woman and the fetus, a large number of pregnant women, as well as medical personnel involved in controlling their asthma, approach asthma treatment during pregnancy cautiously, thereby predisposing the occurrence of acute exacerbations.

The treatment of asthma exacerbations during pregnancy is based on consensus, as pregnant women are generally not included in randomized clinical trials. It is recommended to use the same medications as asthma exacerbations outside of pregnancy, although certain ICSs, such as budesonide, have a safer profile during pregnancy [1]. Additionally, to prevent fetal hypoxia, intensified therapy with SABA, LABA, oxygen (aiming for peripheral blood oxygen saturation above 95%), and early administration of systemic corticosteroids (either orally or intravenously) is recommended during acute asthma exacerbations. Short-term use of systemic corticosteroids (usually oral prednisolone 40 mg daily for five days) is necessary during the treatment of acute asthma exacerbations in pregnancy, and the benefits of their use significantly outweigh the potential risks to the fetus (such as congenital cleft lip and palate, preterm birth, low birth weight) [6,26]. Continuous monitoring of peripheral blood oxygen saturation in the pregnant woman and fetal heart rate is essential [11].

During delivery, it is recommended that the pregnant woman continue her usual asthma maintenance medication, with additional medication for symptom relief as needed. Although acute exacerbations during labor are not common, hyperventilation can lead to bronchospasm and the need for additional SABA inhalations. If high doses of SABA have been used within 48 h before labor, neonatal hypoglycemia can occur, so frequent glucose monitoring in the newborn is recommended during the first 24 h after birth [4]. Monitoring of the pregnant woman during delivery should include regular assessments of respiratory status, oxygen saturation, and symptoms of asthma exacerbation. Continuous fetal heart rate monitoring is also crucial to detect any signs of distress that may be related to maternal asthma or labor complications. Prostaglandin E2 and oxytocin (used for labor induction) can be safely administered to pregnant women with asthma, while derivatives of prostaglandin F2α should be used cautiously in cases of prolonged postpartum bleeding, as they can cause bronchoconstriction [6].

## 5. Conclusions

Managing asthma during pregnancy is a complex task that requires careful consideration of both maternal and fetal health. The current GINA recommendations provide a comprehensive framework for healthcare providers to ensure that pregnant women with asthma receive the best possible care. Scientific research supports the use of asthma maintenance medications and regular monitoring to achieve and maintain asthma control. It is important to emphasize preconception counseling, as it helps ensure that women with asthma are informed about the importance of continuing their therapies once they become pregnant, thus avoiding discontinuation out of misguided fears. By following evidence-based guidelines and individualizing care, healthcare professionals can help pregnant women with asthma enjoy a healthy pregnancy and deliver a healthy baby. It is essential for healthcare providers and expectant mothers to work together to ensure that asthma does not compromise the well-being of either the mother or the child. In addition to the current categorization of asthma medications during pregnancy, we recognize the need for including newer medications, including biological therapy, in future classifications.

## Figures and Tables

**Table 1:** Safety effects of asthma medications used during pregnancy (retrieved and adapted from Middleton et al. [26]. 2020, European Respiratory Society).

Medicine	First Trimester	Second and Third Trimester	Delivery	Lactation
SABA				
salbutamol	compatible	compatible	compatible	compatible
terbutaline	probably safe	probably safe	probably safe	probably safe
LABA				
formoterol	probably safe	probably safe	probably safe	probably safe
salmeterol	probably safe	probably safe	probably safe	probably safe
vilanterol	possibly safe	possibly safe	possibly safe	possibly safe
olodaterol	possibly safe	possibly safe	possibly safe	possibly safe
LAMA				
tiotropium-bromide	possibly safe	possibly safe	possibly safe	compatible
METHYLXANTHINES				
theophylline	compatible	compatible	compatible	compatible
ICS				
budesonide	compatible	compatible	compatible	compatible
beclomethasone	compatible	compatible	compatible	compatible
fluticasone	compatible	compatible	compatible	compatible
triamcinolone	compatible	compatible	compatible	compatible
ciclesonide	probably safe	probably safe	probably safe	probably safe
mometasone	probably safe	probably safe	probably safe	probably safe
SCS				
prednisolone	possibly safe	possibly safe	possibly safe	possibly safe
prednisone	possibly safe	possibly safe	possibly safe	possibly safe
hydrocortisone	possibly safe	possibly safe	possibly safe	possibly safe
LTRA				
montelukast	probably safe	probably safe	probably safe	probably safe
BIOLOGICS				
omalizumab	probably safe	probably safe	probably safe	probably safe
benralizumab	possibly safe	possibly safe	possibly safe	possibly safe
dupilumab	possibly safe	possibly safe	possibly safe	possibly safe
mepolizumab	possibly safe	possibly safe	possibly safe	possibly safe
reslizumab	possibly safe	possibly safe	possibly safe	possibly safe
OTHER				
immunotherapy	probably safe	probably safe	probably safe	probably safe
H1-antagonists	compatible	compatible	probably safe	probably safe
nasal steroids	probably safe	probably safe	probably safe	compatible

Legend: SABA—short-acting beta-2 agonists, LABA—long-acting beta-2 agonists, LAMA—long-acting muscarinic antagonists, ICS—inhaled corticosteroids, SCS—systemic corticosteroids, LTRA—leukotriene receptor antagonists.

## Data Availability

No new data were created or analyzed in this study. Data sharing is not applicable to this article.
